# Seven weeks of Western diet in apolipoprotein-E-deficient mice induce metabolic syndrome and non-alcoholic steatohepatitis with liver fibrosis

**DOI:** 10.1038/srep12931

**Published:** 2015-08-11

**Authors:** Robert Schierwagen, Lara Maybüchen, Sebastian Zimmer, Kanishka Hittatiya, Christer Bäck, Sabine Klein, Frank E. Uschner, Winfried Reul, Peter Boor, Georg Nickenig, Christian P. Strassburg, Christian Trautwein, Jogchum Plat, Dieter Lütjohann, Tilman Sauerbruch, Frank Tacke, Jonel Trebicka

**Affiliations:** 1 Department of Internal Medicine I, University of Bonn, Germany; 2 Department of Internal Medicine II, University of Bonn, Germany; 3 Institute of Pathology, University of Bonn, Germany; 4 Department of Internal Medicine III, RWTH University of Aachen, Germany; 5 Division of Nephrology, RWTH University of Aachen, Germany; 6 Institute of Pathology, RWTH University of Aachen, Germany; 7 Department of Human Biology, University of Maastricht, Netherlands; 8 Institute of Clinical Chemistry and Clinical Pharmacology, University of Bonn, Germany

## Abstract

Non-alcoholic steatohepatitis (NASH) is characterised by hepatic steatosis, inflammation and fibrosis, which might progress to cirrhosis. Human NASH is associated with metabolic syndrome (MS). Currently, rodent NASH models either lack significant fibrosis or MS. ApoE^−/−^ mice are a MS model used in cardiovascular research. The aim of this work was to establish and characterise a novel mouse NASH model with significant fibrosis and MS. ApoE^−/−^ and wild-type mice (wt) were fed either a western-diet (WD), methionine-choline-deficient-diet (MCD) or normal chow. Liver histology, RT-PCR, hepatic hydroxyproline content, triglycerides and cholesterol levels, and fasting glucose levels assessed hepatic steatosis, inflammation and fibrosis. Further, portal pressure was measured invasively, and kidney pathology was assessed by histology. ApoE^−/−^ mice receiving WD showed abnormal glucose tolerance, hepatomegaly, weight gain and full spectrum of NASH including hepatic steatosis, fibrosis and inflammation, with no sign of renal damage. MCD-animals showed less severe liver fibrosis, but detectable renal pathological changes, besides weight loss and unchanged glucose tolerance. This study describes a murine NASH model with distinct hepatic steatosis, inflammation and fibrosis, without renal pathology. ApoE^−/−^ mice receiving WD represent a novel and fast model with all characteristic features of NASH and MS well suitable for NASH research.

Non-alcoholic fatty liver disease (NAFLD) covers a wide spectrum of diseases ranging from mere steatosis to non-alcoholic stetatohepatitis (NASH) with possible progression to cirrhosis^1^. NAFLD is characterised by the metabolic syndrome, dyslipidemia, insulin resistance and obesity^2^,^3^, and associated with increased liver-related mortality^4^. The pathogenesis of NAFLD is poorly understood. Besides the “two-hit”-model which states that after the first damage (steatosis), further damage, such as oxidative stress and subsequent inflammation, leads to NASH^5^, several additional hypotheses on initiation and development of NAFLD were published, which in summary are all characterised by the presence of steatosis, inflammation and fibrosis^6^,^7^,^8^,^9^. Although different animal models show features of NASH, many models lack key features of human NASH like MS (e.g. in the MCD model) or liver fibrosis^10^. Therefore, further animal models especially exhibiting the metabolic syndrome are necessarily needed^11^.

The Apolipoprotein E (ApoE) is an important player in the lipoprotein metabolism in humans and mice. Its absence predisposes to hypercholesterolemia, atherosclerosis and obesity^12^,^13^. Furthermore, it has been published that ApoE^−/−^ mice show spontaneously increased inflammation and high cholesterol levels compared to wt mice^14^. In ApoE^−/−^ mice fed with high-fat-diet (HFD) these effects were even more pronounced and MS develops^15^,^16^,^17^. Long term feeding of western diet (WD) in ApoE-LDLR double deficient mice leads, besides steatosis and inflammation, to liver fibrosis, but also to hepatic tumorigenesis^18^.

Since ApoE deficiency seems to play an important role in MS and might be associated with NAFLD, this study aimed to establish and characterise a rapid NASH model in mice, which mimics major characteristics of human NASH including steatosis, inflammation and fibrosis more closely than existing NASH models.

## Results

### General characteristics

**Figure 1:**
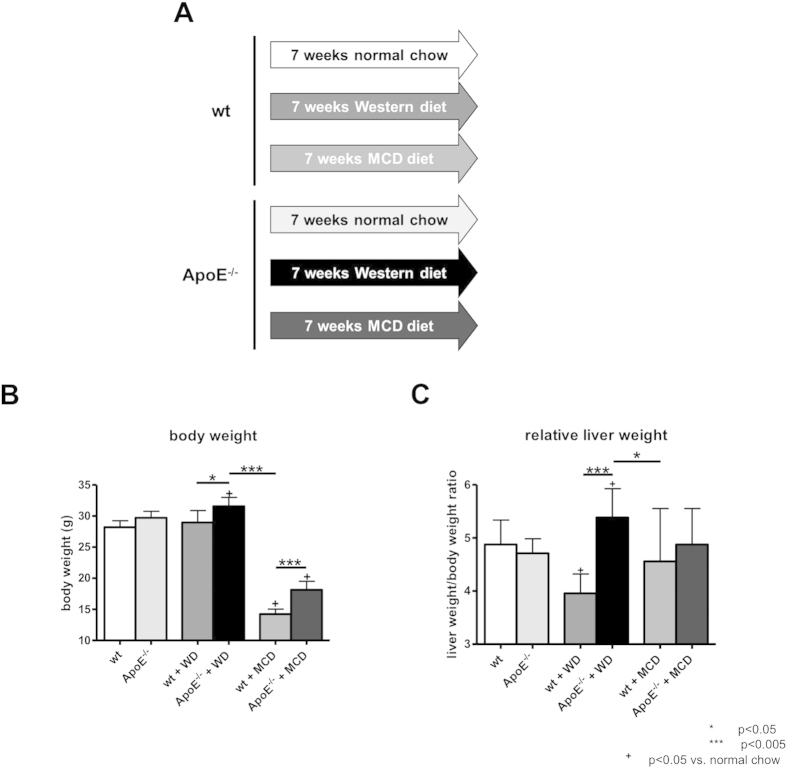
Study design and general characteristics. (**A**) Schematic overview of the study design. wt and ApoE^−/−^ mice were fed either with normal chow, high-fat Western diet (WD) rich in cholesterol or methionine and choline deficient diet (MCD) for seven weeks. (**B**) Body weight of animals before they were sacrificed. ApoE^−/−^ mice fed with WD showed a significant increase in body weight, while mice fed with MCD diet showed a decrease in body weight. (**C**) Relative liver weight (in % of body weight) in ApoE^−/−^ mice fed with WD was increased, suggesting hepatomegaly, whereas it was not altered in mice fed with MCD diet. Graphs are expressed as means ± standard deviation. p < 0.05 was considered significant.

The body weight of mice fed with WD was not significantly increased compared to wt littermates fed with normal chow (Fig. 1B). ApoE^−/−^ mice fed with WD showed a significant increase in mean body weight during the seven weeks of diet compared to ApoE^−/−^ mice fed with normal chow (+ 6,3%). In contrary, both wt and ApoE^−/−^ mice fed with MCD diet showed a significant decrease in mean body weight compared to littermates fed with normal chow, with wt mice showing a more pronounced weight loss comprared to ApoE^−/−^. WD, but not MCD diet, induced a significant increase in the ratio of liver to body weight in ApoE^−/−^ mice (Fig. 1C).

### Hepatic steatosis and cellular ballooning

**Figure 2:**
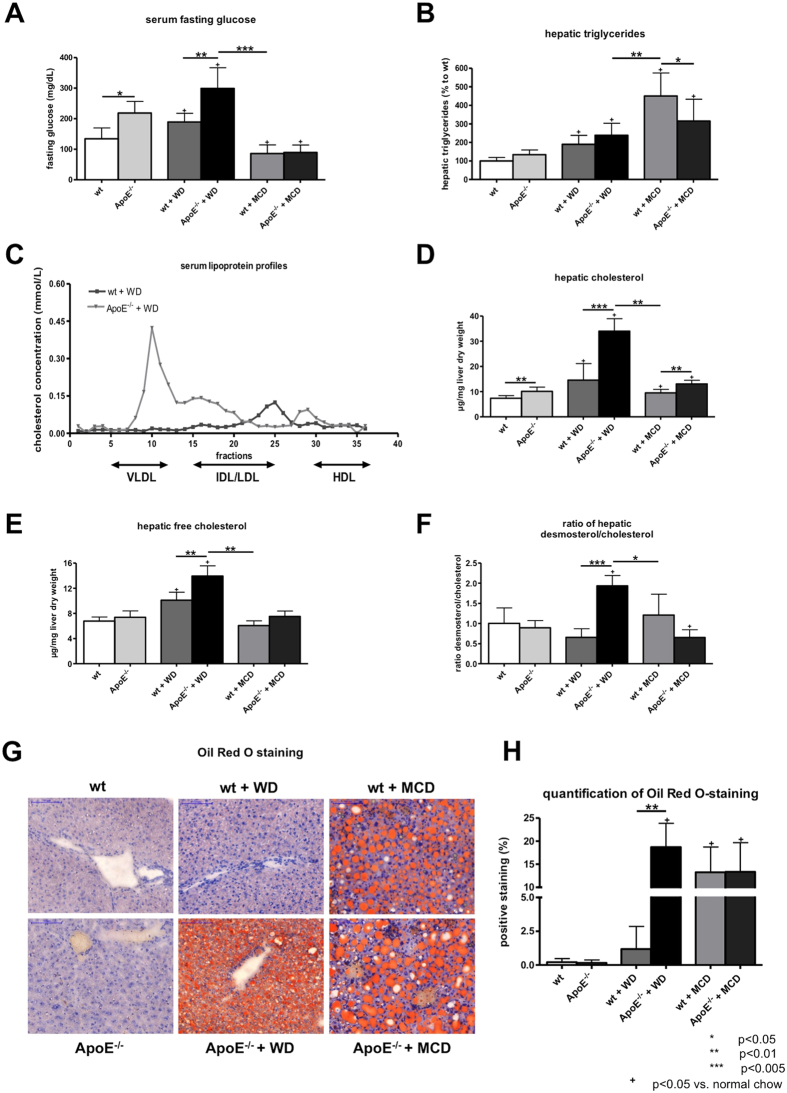
Hepatic steatosis in wt and ApoE^−/−^ mice with or without WD or MCD diet. (**A**) Levels of fasting glucose. ApoE^−/−^ mice fed with Western Diet (WD) showed abnormal fasting glucose levels compared to animals fed with normal chow or methionine and choline deficient (MCD) diet. (**B**) Hepatic triglyceride content expressed as percentage of wt mice. Levels of hepatic triglycerides were increased in ApoE^−/−^ mice fed with WD compared to mice fed with normal chow, but lower than in mice fed with MCD diet. (**C**) Serum lipoprotein profiles of mice fed WD. ApoE^−/−^ mice fed with WD showed shift in serum lipoprotein profile with high amount of very low density lipoproteins (VLDL) compared to wt. Levels of total (**D**) and free (**E**) hepatic cholesterol. ApoE^−/−^ fed with WD showed increased levels of total and free hepatic cholesterol in comparison to the other observed groups. (**F**) Desmosterol/cholesterol ratio was significantly increased in ApoE^−/−^ mice compared to the other observed groups. (**G**) Representative sections and (**H**) quantification (% of positive stained area) of Oil Red O histological staining, as marker of fat accumulation in the liver. ApoE^−/−^ mice fed WD showed significantly higher Oil Red O positive staining than wt mice. In mice fed with MCD diet slightly less Oil Red O positive staining was observed. Graphs are expressed as means± standard deviation. The scale bar is 100 μm. p < 0.05 was considered significant.

Levels of fasting glucose were significantly elevated in ApoE^−/−^ mice compared to wt littermates with normal chow (Fig. 2A). This increase was also significant in mice fed with WD, however with higher levels of serum fasting glucose compared to mice fed with normal chow. By contrast, mice fed with MCD diet for seven weeks showed significantly decreased levels of serum fasting glucose (Fig. 2A).

Hepatic triglycerides were increased in mice fed with WD compared to mice fed with normal chow, with only a slight, but not significant, trend towards higher levels in ApoE^−/−^ mice (Fig. 2B). In mice fed with MCD diet were measured the highest levels of hepatic triglycerides (Fig. 2B). The serum lipoprotein profiles showed profound changes between ApoE^−/−^ mice and wt mice fed with WD, with a marked shift towards increased VLDL levels in ApoE^−/−^ mice fed with WD (Fig. 2C). ApoE^−/−^ mice fed with WD experienced an increase in levels of total and free hepatic cholesterol compared to all other observed groups (Fig. 2D,E). Components of cholesterol biosynthesis, as exemplary shown by total levels of desmosterol, were significantly increased in ApoE^−/−^ mice fed with WD compared to wt littermates (Fig. 2F). According to these data also hepatic cholesterol esters were increased in ApoE^−/−^ mice compared to respective wt littermates with highest levels of hepatic cholesterol esters in ApoE^−/−^ mice fed WD (Supporting Figure S1A). MCD fed wt mice showed also increased levels of hepatic cholesterol esters compared to wt mice fed normal chow, but failed to reach levels of ApoE^−/−^ mice fed WD (Supporting Figure S1A).

Hepatic steatosis was further quantified by Oil Red O staining, a histological marker for accumulation of fat in hepatocytes (Fig. 2G,H). Comparing all groups, ApoE^−/−^ mice fed with WD showed the highest amount of Oil Red O positive staining (Fig. 2G,H). Compared to mice with normal chow, mice fed with MCD diet had significantly increased Oil Red O staining, however no differences between wt and ApoE^−/−^ mice were found (Fig. 2G,H). Interestingly, in ApoE^−/−^ fed with WD microvesicular steatosis was predominant, while in mice fed with MCD diet mainly macrovesicular steatosis was observed (Fig. 2G).

Hepatocyte ballooning, as an important feature for the diagnosis of NASH, was observed in ApoE^−/−^ mice fed WD and in mice fed with MCD diet. Interestingly, in ApoE^−/−^ mice fed normal chow and WD cells were more inflated than in wt mice with respective diet, whereas there was no obvious difference in cellular ballooning in mice fed with MCD diet (Supporting Figure S1B).

### Hepatic inflammation

**Figure 3:**
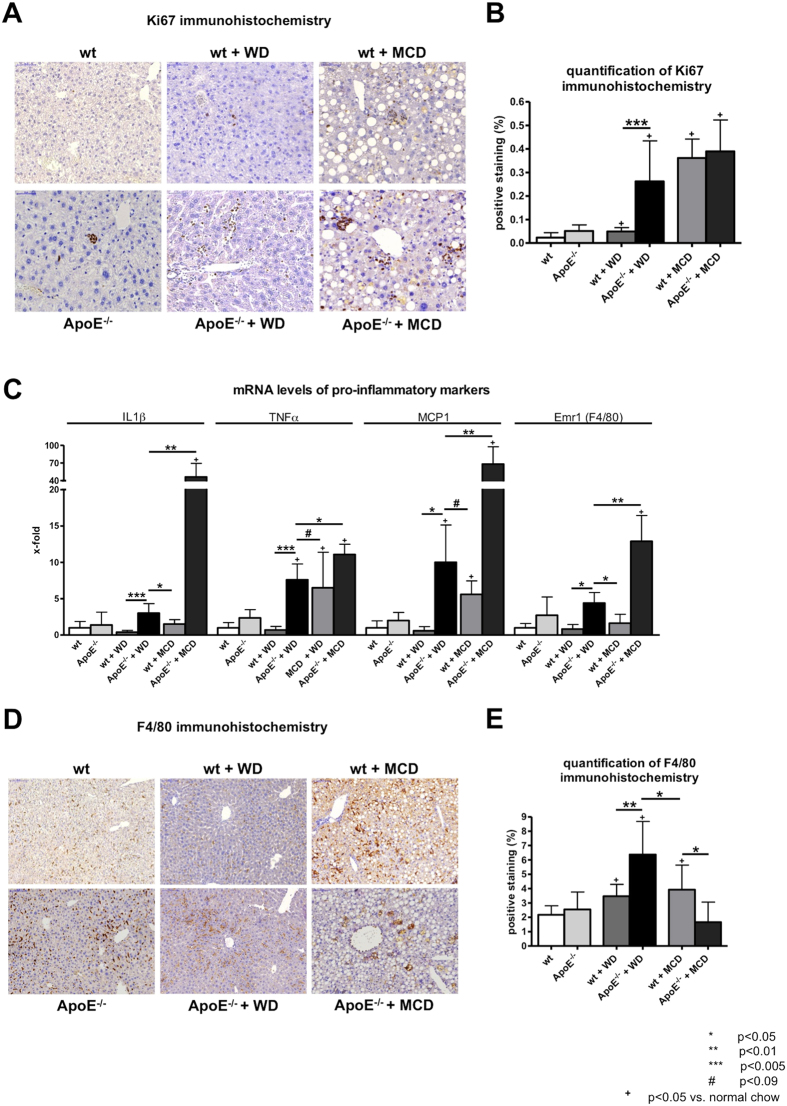
Hepatic inflammation in wt and ApoE^−/−^ mice with or without WD or MCD diet. (**A**) Representative sections and (**B**) quantification (% of positive stained area) of Ki67 immunohistochemistry, as marker of proliferation in the liver. Proliferation was significantly increased in ApoE^−/−^ mice fed with Western diet (WD) compared to wt littermates. In methionine and choline deficient (MCD) diet fed mice proliferation was slightly increased compared to ApoE^−/−^ mice fed with WD. (**C**) mRNA levels of pro-inflammatory markers IL1β, TNFα, MCP and Emr1 (F4/80) were elevated in ApoE^−/−^ mice compared to wt mice fed with WD or MCD diet. ApoE^−/−^ mice fed with MCD diet showed marked elevation of proinflammatory markers on mRNA level. (**D**) Representative sections and (**E**) quantification (% of positive stained area) of F4/80 immunohistochemistry, as marker of macrophages and liver inflammation. ApoE^−/−^ mice fed with WD showed significantly increased levels of F4/80 positive staining compared to wt mice fed with WD and to mice fed with MCD diet. Graphs are expressed as means ± standard deviation. The scale bar is 50 μm for Ki67 respectively 100 μm for F4/80 immunohistochemistry. p < 0.05 was considered significant.

Western diet significantly increased proliferation of inflammatory cells after injury in ApoE^−/−^ mice compared to wt mice, as quantified by Ki67 positive staining (Fig. 3A,B). Levels of Ki67 positive cells in mice fed with MCD diet were only slightly, but not significantly increased, comparable to those of ApoE^−/−^ mice fed with WD. No difference between wt and ApoE^−/−^ mice was found (Fig. 3A,B).

The expression of the proinflammatory markers IL1β, TNFα, MCP1 and Emr1 (F4/80) were significantly higher in ApoE^−/−^ mice fed with WD compared to wildtype mice either fed with WD or with MCD diet (Fig. 3C). Apart from this, ApoE^−/−^ mice fed with MCD showed abnormally high expression levels of pro-inflammatory markers IL1β and MCP1 (Fig. 3C).

Analogous to gene expression levels of Emr1 (F4/80), ApoE^−/−^ mice fed with WD showed significantly increased F4/80 positive staining compared to wildtype mice fed either WD or MCD diet (Fig. 3D,E), indicating an increased recruitment of macrophages. MCD diet in wt mice showed also significantly increased macrophage recruitment compared to wt mice fed normal chow, but failed to reach the levels of ApoE^−/−^ mice fed WD (Fig. 3D,E).

### Liver fibrosis

**Figure 4:**
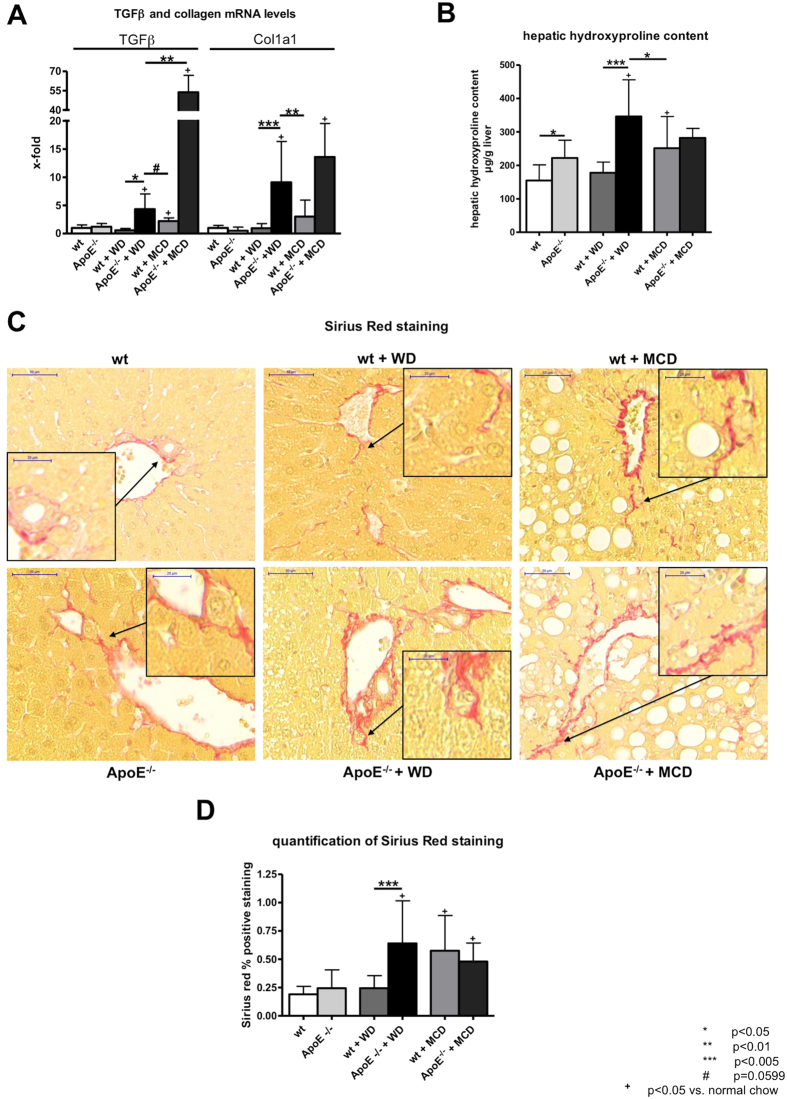
Liver fibrosis in wt and ApoE^−/−^ mice with or without WD or MCD diet. (**A**) Gene expression of profibrotic markers TGFβ and collagen 1 (Col1A), as a marker of hepatic collagen deposition and liver fibrosis. Levels of profibrotic markers were elevated in ApoE^−/−^ fed with Western Diet (WD) compared to wt mice fed with either WD or methionine and choline deficient (MCD) diet. ApoE^−/−^ mice fed with MCD diet showed marked elevation in levels of profibrotic markers on mRNA level. (**B**) Hepatic hydroxyproline content as a marker of fibrosis was significantly elevated in ApoE^−/−^ mice fed with WD compared to wt mice either fed with WD or MCD diet. (**C**) Representative sections and (**D**) quantification (% of positive stained area) of Sirius red histological staining. ApoE^−/−^ mice fed with WD showed significantly more Sirus red positive staining than wt mice fed with WD. In mice fed with MCD diet Sirius red positive staining was comparable to ApoE^−/−^ mice fed with WD. Graphs are expressed as means ± standard deviation. The scale bar is 50 μm respectively 20 μm for the magnifications. p < 0.05 was considered significant.

Liver fibrosis was assessed by hepatic collagen deposition (hepatic hydroxyproline content, collagen mRNA), TGFβ mRNA expression and Sirius red staining. Gene expression of collagen type I (Col1a1) and profibrotic marker TGFβ were increased in ApoE^−/−^ mice fed with WD compared to wt mice fed either with WD or with MCD diet (Fig. 4A). Similarly to mRNA levels of proinflammatory markers, the expression levels of collagen and TGFβ were highest in ApoE^−/−^ mice fed with MCD (Fig. 4A).

The hepatic hydroxyproline content was significantly increased in ApoE^−/−^ mice fed with WD compared to wt mice fed either with WD or to mice fed with MCD diet (Fig. 4B). Quantification of the Sirius red staining supported the finding that fibrosis was significantly increased in ApoE^−/−^ mice fed with WD compared to wt littermates (Fig. 4C,D). The staining showed fibrosis in ApoE^−/−^ mice fed with WD, which could not be observed in mice fed normal chow and wt mice fed WD (Fig. 4C). In mice fed with MCD diet levels of Sirius red positive staining were comparable to those in ApoE^−/−^ mice fed with WD (Fig. 4C,D). Analysing the fibrosis areas separately, periportal and lobular fibrosis were more pronounced in MCD fed mice and in ApoE^−/−^ mice fed WD (Table 1).

### Hepatic stellate cell activation and portal pressure

**Figure 5:**
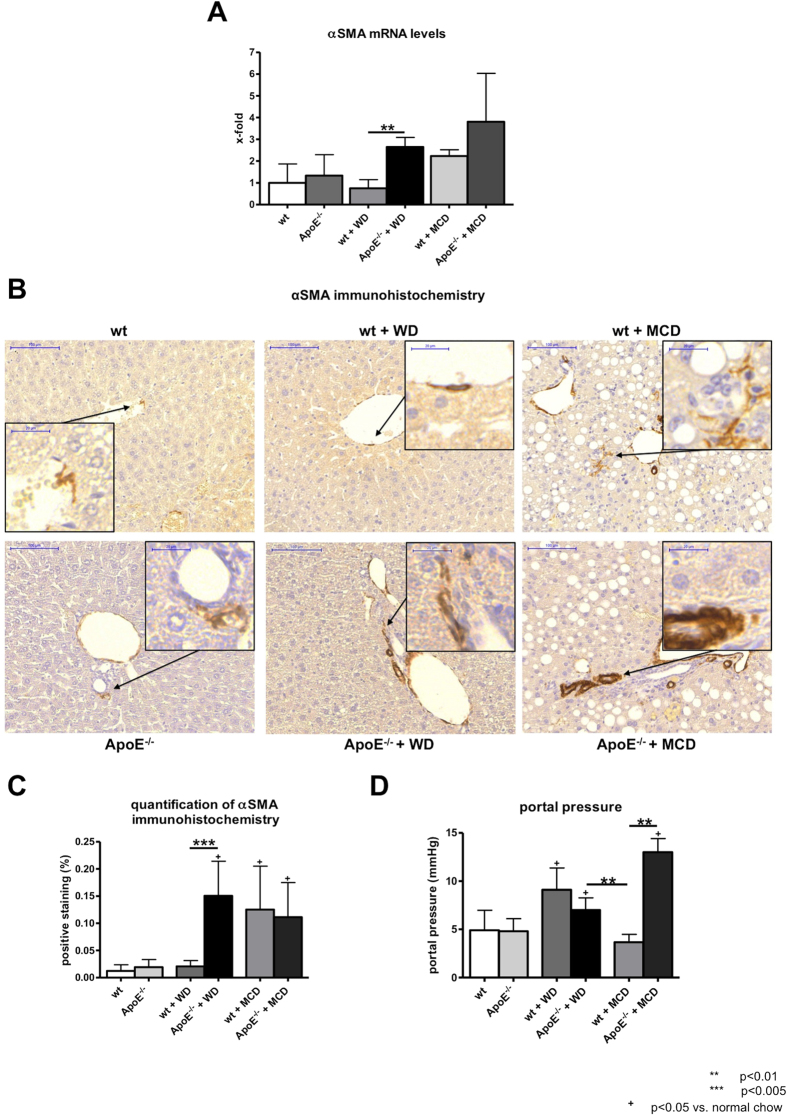
Hepatic stellate cell activation in wt and ApoE^−/−^ mice with or without WD or MCD diet. (**A**) Gene expression of αSMA, as a surrogate marker for hepatic stellate cell activation showed increased expression in ApoE^−/−^ mice fed with Western Diet (WD) compared to their wt littermates. Expression levels of αSMA in mice fed with methionine and choline deficient (MCD) diet were comparable to those in ApoE^−/−^ mice fed with WD. (**B**) Representative sections and (**C**) quantification (% of positive stained area) of αSMA histological staining. ApoE^−/−^ mice fed with WD showed significantly more αSMA positive staining than wt mice fed with WD. In mice fed with MCD diet αSMA positive staining was comparable to ApoE^−/−^ mice fed with WD. (**D**) Invasive portal pressure measurement showed increased portal pressure in ApoE^−/−^ mice fed with WD compared to wt mice fed with MCD diet. ApoE^−/−^ mice fed MCD diet showed abnormal high portal pressure. Graphs are expressed as means ± standard deviation. The scale bar is 100 μm respectively 20 μm for the magnifications. p < 0.05 was considered significant.

Activation of hepatic stellate cells was assessed by mRNA levels and immunohistochemical staining of the surrogate marker αSMA. In ApoE^−/−^ mice fed with WD mRNA levels of αSMA were significantly elevated in comparison with wt littermates (Fig. 5A). In mice fed with MCD diet gene expression levels of αSMA were not significantly different from those of ApoE^−/−^ mice fed with WD. Corresponding to the mRNA levels αSMA positive staining was significantly increased in ApoE^−/−^ mice fed with WD compared to wt mice and similar in comparison to mice fed with MCD diet (Fig. 5B,C). In ApoE^−/−^ mice fed with WD the portal pressure was increased after seven weeks of diet compared to ApoE^−/−^ mice fed normal chow and to wt mice fed with MCD diet. Interestingly, wt mice fed MCD diet showed increased fibrosis compared to wt fed with normal chow, but failed to develop increased portal pressure. In contrast, wt mice fed WD and ApoE^−/−^ mice fed MCD diet showed higher portal pressure than ApoE^−/−^ mice fed WD (Fig. 5D).

### Renal pathology

**Figure 6:**
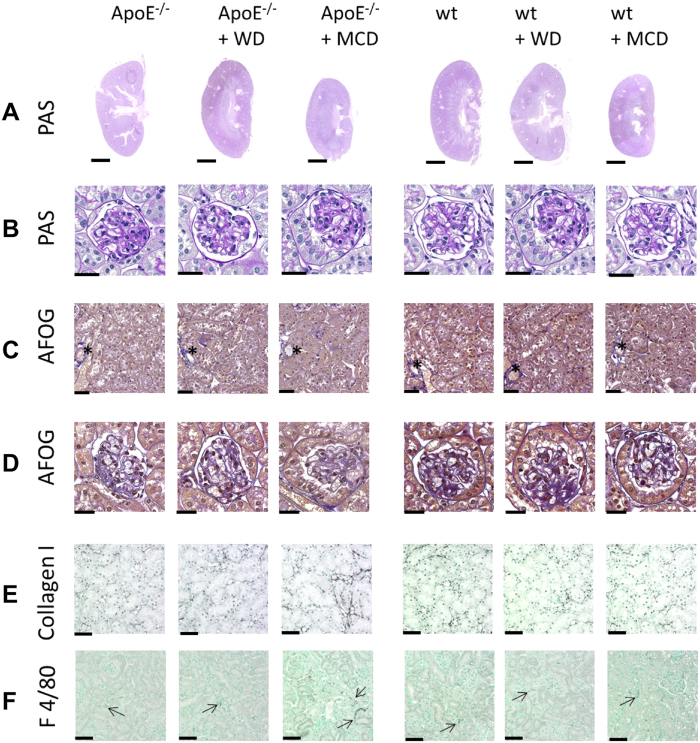
Kidney pathology in wt and ApoE^−/−^ mice with or without WD or MCD diet. Kidney morphology and detailed analyses of (**A**,**B**) Periodic Acid Schiff’s (PAS) and (**C**,**D**) Acidic Fuchsin Orange G (AFOG) stainings did not reveal any obvious pathological alterations in glomeruli (**B**,**D**), tubulointerstitium (**C**) or vessels (asterixes in **C**). No macroscopic differences were observed between the groups except of smaller kidney size in mice fed with MCD diet. (**E**) Immunohistochemistry for collagen type I, as a fibrosis marker and (**F**) F4/80, as a marker for renal monocyte/macrophage/dendritic cells showed normal findings comparable to wildtype mice fed with normal chow in all groups, apart from increased inflammation and fibrosis in ApoE^−/−^ mice fed with MCD diet. Scale bars are 2.5 mm for A, 25 μm for B,D and 50 μm for C,E,F.

Macroscopically, no pathological changes where observed in the kidneys apart smaller size of kidneys from mice fed with MCD diet (Fig. 6A). Detailed analyses of Periodic Acid Schiff’s (PAS) and Acidic Fuchsin Orange G (AFOG) stainings did not reveal any obvious pathological alterations in glomeruli (Fig. 6B,D), tubulointerstitium (Fig. 6C) or vessels (Fig. 6C with asterixes).

No pathological infiltrates of F4/80 positive cells were observed in any of the groups apart from a significant increase in F4/80 positive cells in ApoE^−/−^ mice fed with MCD diet (Table 2 and Fig. 6E). Similarly, collagen type I immunohistochemistry confirmed the findings from histology with no observable fibrosis in neither of the groups apart from significantly increased deposition in ApoE^−/−^ mice fed with MCD diet (Table 2 and Fig. 6F).

### Discussion

This study characterises a new mouse NASH model with a clear metabolic syndrome phenotype. ApoE^−/−^ mice fed with high fat western diet (WD), enriched with cholesterol, over seven weeks, developed metabolic syndrome and showed: (i) fast development of steatosis, (ii) hepatocyte ballooning, (iii) high fasting glucose levels, (iv) inflammation, (v) with pronounced fibrosis, (vi) portal hypertension and (vii) no kidney injury.

Many of the currently existing animal models, either genetically or dietary induced, lack to reflect the full spectrum of human NASH^10^,^11^. Since ApoE deficiency might be associated with NAFLD/NASH and seems to play an important role in the development of MS^12^,^13^,^14^,^15^,^16^,^17^, ApoE^−/−^ mice were chosen to establish this novel NASH model. In these mice, western life style was simulated by western diet (WD), rich in cholesterol and fat, enhancing the effects of the knockout and leading to NASH. We directly compared our WD model in ApoE^−/−^ mice to the WD in wt mice as well as to the well-characterised methionine and choline deficient (MCD) diet. Genetically based (ob/ob, db/db) and dietary induced models (MCD, fructose) lead to the development of steatosis. However, ob/ob mice are protected against fibrosis^19^. In contrast to human NASH, steatosis in SREBP transgenic mice and some dietary models (MCD, fructose) is not linked to obesity^20^,^21^,^22^. Furthermore, the metabolic profile of MCD, associated with weight decrease, is the opposite of human NASH. The increase in the liver to body weight ratio in ApoE^−/−^ mice fed with WD demonstrates hepatomegaly, a characteristic, which was not present in mice fed with MCD diet. MCD not only led to decreased body weight, but also to a decreased kidney size, which was more pronounced in the ApoE^−/−^ mice fed with MCD. However, it was also present in wt mice fed with MCD diet. Importantly, ApoE^−/−^ mice fed with MCD diet showed kidney inflammation and fibrosis.

We found increased liver content of triglycerides and hepatic steatosis together with adiposity in ApoE^−/−^ mice fed with WD, a finding well in line with a previous study^15^. Another recent study employed ApoE/LDL-receptor double knock-out mice fed with WD for 35 weeks to induce steatosis, fibrosis and, in about 30% of the animals, liver tumors^18^. However, the deficiency in ApoE seems to be more important in the development of NASH as shown by our results finding the full picture of NASH after only seven weeks of diet.

Compared to the frequently used MCD model, after seven weeks animals on MCD diet did not develop abnormal fasting glucose concentrations, in fact these were significantly reduced compared to mice fed with normal chow. Abnormal fasting glucose levels, reflecting insulin resistance, are an important pathophysiological characteristic for the development of human NASH, which were found in ApoE-deficient mice after seven weeks of WD.

Another interesting finding resembling human NASH was the increase in free hepatic cholesterol and in the desmosterol/cholesterol ratio observed in ApoE^−/−^ mice on WD. An increase in free hepatic cholesterol has been described also in other experimental models of NASH^23^,^24^. In humans free hepatic cholesterol and the hepatic desmosterol/cholesterol ratio are also increased in individuals with NASH^24^,^25^, suggesting that dysregulation of lipid metabolism in ApoE^−/−^ mice fed with WD is similar to human NASH. Wt mice fed a WD and ApoE^−/−^ mice under normal chow did not develop hepatic steatosis and adiposity, while ApoE^−/−^ mice under WD did. Moreover, the ApoE^−/−^ mice on WD showed a clear dyslipidemia characterised by elevated serum VLDL and LDL cholesterol concentrations. Interestingly, MCD fed animals showed relatively high levels of triglycerides, but low levels of total and free hepatic cholesterol, which was the opposite of what was observed in ApoE^−/−^ mice fed with WD. This and the type of steatosis, microvesicular in mice fed WD and macrovesicular in mice fed MCD diet, showed that composition and development of steatosis differ substantially between these two diets.

Steatosis and inflammation develop in several existing animal models, when the genetic model is combined with a specific diet^11^,^16^,^17^,^26^. Similarly, ApoE^−/−^ mice developed inflammation, assessed by increased IL1β mRNA level and macrophage recruitment, after high fat cholesterol-rich western diet, which is similar to humans.

A further advantage of this new model is the rapid development of NASH within seven weeks of diet. In contrast, other dietary NASH models require a minimum of 15 weeks of diet or even longer induction time^27^,^28^,^29^,^30^,^31^. Therefore, our model offers an acceptable time frame and decreases animal cost. APOE2ki mice, in comparison, show only acute effect on hepatic inflammation and liver injury, which, however, resolves under long-term feeding of high-fat diet^29^,^32^.

ApoE^−/−^ mice fed with WD were comparable to the established MCD model with respect to liver inflammation and liver fibrosis. The combination of ApoE^−/−^ mice and MCD diet showed comparable results regarding inflammation and fibrosis to MCD fed wt mice and ApoE^−/−^ mice fed WD, but lacks the same major characteristics as the established NASH model of MCD fed wt mice and further showed renal pathologies. This leads to the assumption that the proper combination of genetic and dietary factors was responsible for the observed phenotype of NASH in MS.

Additionally, ApoE^−/−^ mice fed with WD developed significant fibrosis, indicated by upregulation of mRNA levels of collagen and cytokines (TGFβ), increased formation of hepatic collagen together with activation of hepatic stellate cells. Furthermore, portal pressure was increased in ApoE^−/−^ mice fed with WD compared to ApoE^−/−^ mice fed normal chow and wt mice fed with MCD diet, a phenomenon also found in humans^33^. Interestingly, development of fibrosis in MCD fed mice was comparable to ApoE^−/−^ mice fed WD, but wt mice fed MCD diet showed no increase in portal pressure. The role of ApoE^−/−^ itself on vascular alterations following increase of portal hypertension cannot be excluded. However, the more pronounced fibrosis in ApoE^−/−^ mice fed WD suggests fibrosis as a major factor leading to portal hypertension.

In other experimental NASH models pronounced liver fibrosis developed only after long-term feeding of diet and then with a high incidence of liver tumors and HCC^18^,^30^,^31^,^34^,^35^. The occurrence of liver tumors and HCC in murine NAFLD/NASH models seems to be increased in models with long-term feeding and independent of the composition of the diet containing high-fat and independent of the genetic background^18^,^34^,^35^. Therefore a NASH model with a short period of feeding, as it is described in this study, could be helpful.

The lack of hepatic fibrosis is a major weakness of many existing NASH models^11^, which is present in the described model of the current study. Interestingly, especially in the MCD model, which is a widely used model, after seven weeks of diet the proinflammatory markers are significantly lower in wt than in ApoE^−/−^ mice under MCD diet. But even though MCD diet seems to be a major proinflammatory agent in the liver, since ApoE^−/−^ mice fed MCD diet show a huge mRNA upregulation of proinflammatory and profibrotic markers, these markers were similar on protein level between mice fed with WD and MCD diet.

In summary, the present study described a novel, reliable and fast murine NASH model with metabolic syndrome showing major characteristics of human NASH such as distinct hepatic steatosis, inflammation, fibrosis and increase in portal pressure. At the same time this novel NASH model is an economic and rapid novel NASH model. This model is comparable with the established MCD model regarding hepatic steatosis, inflammation and fibrosis, but has additional advantages including increased body weight and metabolic syndrome, which are not present in the MCD model. ApoE^−/−^ mice fed with WD could be a novel and valuable tool in NASH research.

## Material and Methods

### Animals

Twelve-week-old wildtype mice (wt, C57BL/6J background; Charles River, Wilmington, USA) and ApoE-knockout mice (ApoE^−/−^, C57BL/6J background; Charles River, Wilmington, USA) were used. All mice received water and chow *ad libitum*. The animals were kept at 22 °C with a 12 hours day/night cycle. Mice were either fed a normal chow, MCD diet (Ssniff, Soest, Germany) or a high-fat, cholesterol-rich diet (western diet; WD) containing 21% fat (with coconut oil), 19.5% casein and 1.25% cholesterol (Ssniff, Soest, Germany) for seven weeks (Fig. 1A) as described previously^36^,^37^,^38^. No difference in food intake was noticed. All experiments were performed in accordance to the “German Animal-Protection Law” and the guidelines of the animal care unit at our university (Haus für experimentelle Therapie, Bonn, Germany) and approved by the relevant North Rhine-Westphalian state agency for Nature, Environment and Consumer Protection (LANUV, Germany) under the file reference LANUV84-02.04.2014.A137.

### Hemodynamic studies

Hemodynamic studies were performed as described previously^39^. Briefly, mice were fasted overnight with free access to water. Animals were anaesthetised using ketamine/xylazine (125 mg ketamine hydrochloride/kg body weight and 17 mg xylazine/kg body weight s.c.). After median laparotomy a PE-50 catheter was introduced into a small ileocolic vein and advanced to portal vein for the measurement of portal pressure. The catheter was connected to a pressure transducer (Hugo Sachs Elektronik, March-Hugstetten, Germany) for the measurements. After insertion of the catheter, animals were allowed to hemodynamically stabilise.

### Tissue collection and biochemical analysis

After 7 weeks of diet, the mice were anesthetised and laparotomy was performed for tissue collection. The liver was cut into fragments. Liver samples were either snap-frozen in liquid nitrogen and stored at −80 °C or fixed in formaldehyde (4%) as described previously^40^,^41^,^42^. Kidney samples were fixed in methyl Carnoy’s solution. Fasting glucose was measured using Akku-Chek Aviva (Roche Diagnostics, Mannheim, Germany).

### Hepatic hydroxyproline and triglyceride content

Hepatic hydroxyproline was photometrically measured in liver hydrolysates as previously described^40^,^42^. Analogue segments (200 mg) of snap-frozen livers were first hydrolysed in HCl (6N) at 110 °C for 16 hours and then filtered and aliquoted. Aliquots (50 μl) were incubated with chloramine T (2,5 mM) for 5 minutes and subsequently with Ehrlich’s reagent (410 mM) for 30 minutes at 60 °C. Adsorption was determined three times at 558 nm after a standard curve for hydroxyproline was compiled. The results are expressed as  μg/g of wet liver tissue. Hepatic triglyceride content was measured from homogenised snap-frozen liver samples using TG liquicolor mono (Human Diagnostics, Wiesbaden, Germany) according to the manufacturer’s instructions, as described previously^38^.

### Quantitative RT-PCR

RNA isolation from snap-frozen liver samples, reverse transcription and detection by RT-PCR were performed as described previously^40^,^43^. The following assays provided by Applied Biosystems (Foster City, USA) were used: Acta2 (αSMA; Mm00725412_s1), col1a1 (collagen-1 ; Mm00801666_g1), Emr1 (F4/80; Mm00802529_m1), Il1b (IL1β; Mm01336189_m1), Ccl2 (MCP1; Mm00441242_m1), Tgfb1 (TGFβ; Mm03024053_m1) and Tnf (TNFα; Mm00443258_m1). 18S rRNA served as endogenous control. Results were expressed as 2^−ΔΔCt^, and express the x-fold shift of gene transcription compared to the respective control group.

### Histological staining

For detection of fat accumulation in the liver, 6 μm thick sections of snap-frozen liver samples were prepared using a cryostat. Sections were dehydrated overnight, fixed in 10% formalin, washed with 60% Isopropyl alcohol and stained with Oil red O (3%) followed by counterstaining with hematoxylin as described previously^44^,^45^,^46^. To detect collagen fibers, paraffin-embedded sections (2-3 μm) were treated with 0.1% Sirius-red F3B in saturated picric acid (Chroma, Münster, Germany) as described previously^40^,^42^. Paraffin-embedded sections (2–3 μm) were also stained hematoxlin-eosin (H&E) for detection of cellular ballooning as described previously^8^. Immunohistochemical stainings for αSMA, Ki67 and F4/80 were performed in paraffin-embedded sections (2–3 μm). The sections were incubated with a mouse-anti-SMA antibody (Actin clone 1A4; Dako, Hamburg, Germany), rabbit-anti-Ki67 (clone SP6; abcam, Cambridge, UK) or rat-anti-F4/80 (clone BM8; BMA Dianova, Hamburg, Germany). Thereafter, biotinylated goat-anti-mouse (Dako, Hamburg, Germany), goat-anti-rabbit (Dako, Hamburg, Germany) and rabbit-anti-rat (Biozol, Eching, Germany) secondary antibodies were used respectively. Finally, sections were counterstained with hematoxylin. Histological stainings were analysed according to Ishak score^47^ and digitalised using Pannoramic MIDI (3DHistech, Budapest, Hungary). For computational analysis (Histoquant, 3DHistech, Budapest, Hungary) large bile ducts and vessels were excluded, following the principles of computational analysis as described previously^44^,^48^. For distinction of periportal and lobular fibrosis, 5–7 periportal and lobular areas were analysed for positive staining in each section under high magnification.

Histology and indirect immunohistochemistry of kidney samples were performed in paraffin-embedded sections (1 μm) as described previously^49^. In short, renal tissue was stained either with periodic acid Schiff’s (PAS), Acidic Fuchsin Orange G (AFOG) or indirect immunohistochemistry using following primary antibodies: monoclonal rat anti-mouse F4/80 (Serotec AbD, Oxford, UK) and polyclonal goat anti-human collagen type I (Southern Biotechnology Associates, Birmingham, AL, USA) cross reacting with murine collagen type I. The secondary antibodies used were biotinylated and affinity purified (Vector, Burlingame, CA, USA) and negative controls, performed as previously described^49^, remained negative (data not shown). The histomorphology was analysed by an experienced nephropathologist (P.B.). The immunohistochemistry was assessed using computer based morphometry of positively stained cortical area for collagen type I using the ImageJ v1.48 software (http://imagej.nih.gov/ij/) and by manual counting of F4/80 positive cells in 15 cortical high-power fields.

### Determination of hepatic cholesterol, desmosterol and cholesterol in lipoprotein fractions

Total and free hepatic cholesterol and cholesterol esters were determined by gas chromatography-flame ionisation detection. To determine the concentrations of total cholesterol tissue lipid chloroform extracts, containing cholesteryl fatty acid esters and free cholesterol, were completely hydrolysed prior to derivatisation to the corresponding trimethylsilylethers, while free cholesterol was silylated without alkaline hydrolysis of the lipid extracts. The precursor desmosterol as surrogate marker of endogenous cholesterol biosynthesis was detemined by gas chromatography-mass spectrometry (selected ion monitoring)^50^,^51^. Serum lipoprotein profiles were determined by fast protein liquid chromatography (FPLC) as described earlier^52^.

### Statistical analysis

Data are presented as mean ± standard deviation (SD). Group size was at least n = 5 for each group. Statistical analysis of two groups was performed with Mann-Whitney-*U* test respectively Kruskal-Wallis test with Dunn’s post-hoc test for comparisons of more than two groups using GraphPad Prism (La Jolla, CA, USA). *p*-values< 0.05 were considered statistical significant.

## Additional Information

**How to cite this article**: Schierwagen, R. *et al.* Seven weeks of Western diet in apolipoprotein-E-deficient mice induce metabolic syndrome and non-alcoholic steatohepatitis with liver fibrosis. *Sci. Rep.* **5**, 12931; doi: 10.1038/srep12931 (2015).

## Supplementary Material

Supporting figure 1

## Figures and Tables

**Table 1:** Quantification of periportal and lobular fibrosis.

	periportal positive area (%)	lobular positive area (‰)
wt	1.433 ± 0.697	0.17 ± 0.08
ApoE^−/−^	1.728 ± 0.306	0.12 ± 0.09
wt + WD	1.812 ± 1.138	0.16 ± 0.02
ApoE^−/−^ + WD	6.718 ± 3.584^bb,cc^	0.99 ± 0.11
wt + MCD	5.804 ± 1.967^a,c^	0.59 ± 0.06
ApoE^−/−^ + MCD	4.827 ± 1.533^b,c^	0.93 ± 0.10

Quantification of Sirius-red positive stained area, as a marker of hepatic fibrosis, in periportal and lobular areas. ApoE^−/−^ mice fed with Western diet (WD) and mice fed methionine and choline deficient (MCD) diet showed significantly increased periportal fibrosis compared to mice fed normal chow and to wildtype (wt) mice fed with WD. Additionally, in mice with increased periportal fibrosis there is also a trend towards increased lobular fibrosis. Data are presented as mean ± standard deviation. p < 0.05 was considered significant. a = p < 0.05 vs. wt, b = p < 0.05 vs. ApoE^−/−^, bb = p < 0.01 vs. ApoE^−/−^, c = p < 0.05 vs wt + WD, cc = p < 0.01 vs. wt + WD.

**Table 2:** Quantification of renal inflammation and fibrosis.

	F4/80 positive cells	collagen positive area (%)
wt	3.73±1.02	1.47±0.57
ApoE^−/−^	4.60±1.79	1.06±0.44
wt + WD	3.66±0.86	1.47±0.81
ApoE^−/−^ + WD	4.95±1.76	1.41±0.33
wt + MCD	2.76±0.67	1.48±0.49
ApoE^−/−^ + MCD	13.57±3.08	3.25±1.11

Quantification of F4/80 positive cells as a marker for monocytes/macrophages/dendritic cells and inflammation and collagen positive stained area (%), as a marker of renal fibrosis, showed comparable values in all groups, apart from increased inflammation and fibrosis in ApoE^−/−^ mice fed with MCD diet. Data are presented as mean ± standard deviation.
