# Supplementary material for: Seven weeks of Western diet in apolipoprotein-E-deficient mice induce metabolic syndrome and non-alcoholic steatohepatitis with liver fibrosis
Source: Sci Rep. 2015 Aug 11;5:12931. doi: 10.1038/srep12931 (PMC4531783; doi:10.1038/srep12931)
Supplement: Supporting figure 1 [file srep12931-s1.doc]

# Seven weeks of Western diet in apolipoprotein-E-deficient mice induce metabolic syndrome and non-alcoholic steatohepatitis with liver fibrosis.

Robert Schierwagen^1^†, Lara Maybüchen^1^†, Sebastian Zimmer^2^, Kanishka Hittatiya^3^, Christer Bäck^4^, Sabine Klein^1^, Frank E Uschner^1^, Winfried Reul^1^, Peter Boor^5,6^, Georg Nickenig^2^, Christian P Strassburg^1^, Christian Trautwein^4^, Jogchum Plat^7^, Dieter Lütjohann^8^, Tilman Sauerbruch^1^, Frank Tacke^4^, Jonel Trebicka^1^*

**
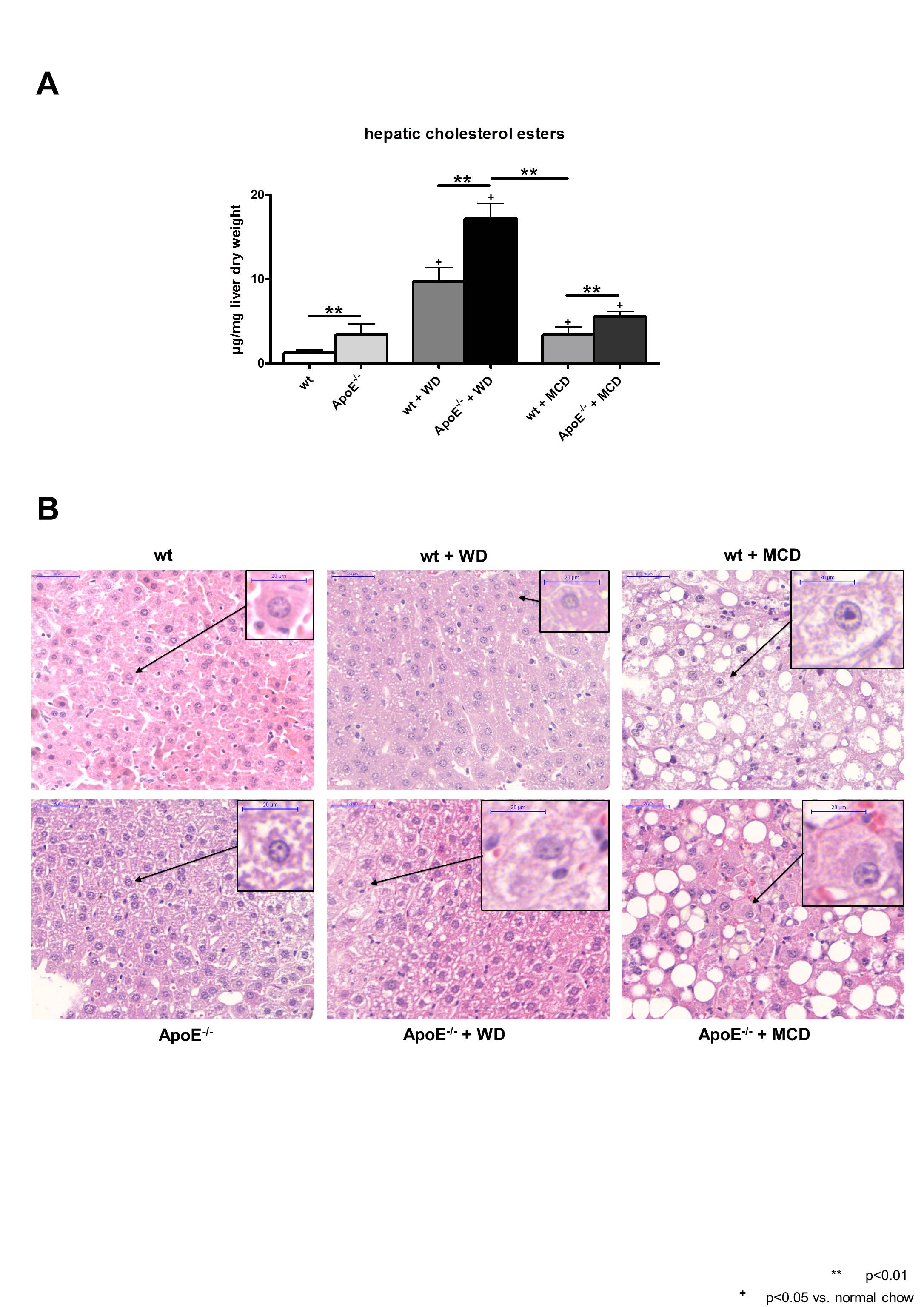
**

**Supporting Figure S1: Hepatic cholesterol esters and cellular ballooning in wt and ApoE^-/-^ mice with or without WD or MCD diet.** (A) Levels of hepatic cholesterol esters showed highest levels in ApoE^-/-^ mice fed WD. Hepatic cholesterol esters were incerased in ApoE^-/-^ mice in each diet compared to wt litermates. MCD fed wt mice showed increased (p<0.0079) levels of hepatic cholesterol esters compared to wt mice fed normal chow. (B) Representative liver histology of H&E stained sections demonstrated occurence of hepatocyte ballooning. Ballooning was observed in ApoE^-/-^ fed Western diet (WD) and in mice fed methionine-choline-deficient (MCD) diet, as shown by exemplary cells in the insets. The scale bar is 50µm respectively 20µm for the insets.
